# Poleward upgliding Siberian atmospheric rivers over sea ice heat up Arctic upper air

**DOI:** 10.1038/s41598-018-21159-6

**Published:** 2018-02-13

**Authors:** Kensuke K. Komatsu, Vladimir A. Alexeev, Irina A. Repina, Yoshihiro Tachibana

**Affiliations:** 1 Faculty of Bioresources, Mie University, Tsu, Japan; 2 International Arctic Research Center, University of Alaska Fairbanks, Fairbanks, USA; 3 Obukhov Institute of Atmospheric Physics, Russian Academy of Sciences, Moscow, Russia; 4 Lomonosov Moscow State University, Moscow, Russia; 5 Hydrometeorological Center, Moscow, Russia

## Abstract

We carried out upper air measurements with radiosondes during the summer over the Arctic Ocean from an icebreaker moving poleward from an ice-free region, through the ice edge, and into a region of thick ice. Rapid warming of the Arctic is a significant environmental issue that occurs not only at the surface but also throughout the troposphere. In addition to the widely accepted mechanisms responsible for the increase of tropospheric warming during the summer over the Arctic, we showed a new potential contributing process to the increase, based on our direct observations and supporting numerical simulations and statistical analyses using a long-term reanalysis dataset. We refer to this new process as “Siberian Atmospheric Rivers (SARs)”. Poleward upglides of SARs over cold air domes overlying sea ice provide the upper atmosphere with extra heat via condensation of water vapour. This heating drives increased buoyancy and further strengthens the ascent and heating of the mid-troposphere. This process requires the combination of SARs and sea ice as a land-ocean-atmosphere system, the implication being that large-scale heat and moisture transport from the lower latitudes can remotely amplify the warming of the Arctic troposphere in the summer.

## Introduction

The fact that the Arctic is heating up faster than the rest of Earth, referred to as Arctic amplification^1,2^, is one of the most significant global environmental concerns. Rapid Arctic warming occurs at the surface^1,3^, and numerous processes have been proposed as the causes of this amplification^2,4^. Whereas the positive feedback between sea-ice reduction and solar absorption seems to be the major contributor to the warming, numerical models in which albedo or sea surface temperature (SST) is kept constant nevertheless predict an amplified warming in the Arctic^5,6^. Arctic amplification occurs not only near Earth’s surface but also throughout the troposphere during all seasons^7,8^. One contributor to the mid-tropospheric warming is the changing poleward heat transport from outside the Arctic^5,7–10^. Moisture is considered to be an important contributor to this enhanced poleward heat transport^5,11,12^ because water vapour absorbs longwave radiation and is the strongest greenhouse gas in the free atmosphere. Water vapour injection also strengthens Arctic amplification at the surface because of the increase of the downward flux of infrared radiation emitted by water vapour^13–15^. Poleward moisture transport is therefore a key process that contributes to surface and mid-tropospheric Arctic warming.

Water vapour transport by transient eddies (i.e., packets of synoptic cyclones) dominates the total transport of water vapour into the Arctic^16,17^. Evaporation from the surface of the North Atlantic and North Pacific Oceans accounts for much of the moisture in this poleward transport. These intensive northward movements of moisture in mid-latitudes have river-like structures and are referred to as Atmospheric Rivers; they carry large amounts of water vapour within narrow bands over oceans^18,19^. The narrow bands form filamentary regions within a warm conveyor belt ahead of the cold front of extratropical cyclones^20,21^. Recent studies of Atmospheric Rivers in the Atlantic and Pacific oceans have indicated that they contribute to the melting of the Greenland ice surface in summer^22^ and Arctic surface warming in winter^14,23^. These two oceanic areas act as an important source of water vapour to the Arctic throughout the year, while North America and Siberia can also play a role as continental source regions in the summer^24^. The atmospheric water vapour and precipitable water over eastern Siberia has been increasing^25^, and a positive precipitation trend has also been observed during the summer^26,27^. In addition to these recent conditions favouring greater precipitation, atmospheric cyclonic circulation over northern Eurasia has strengthened^28^ in association with reductions of sea ice^29^. These previous studies have suggested to us that cyclones from Siberia can transport water vapour to the Arctic and can enhance warming through a certain mechanism during the summer.

**Figure 1:**
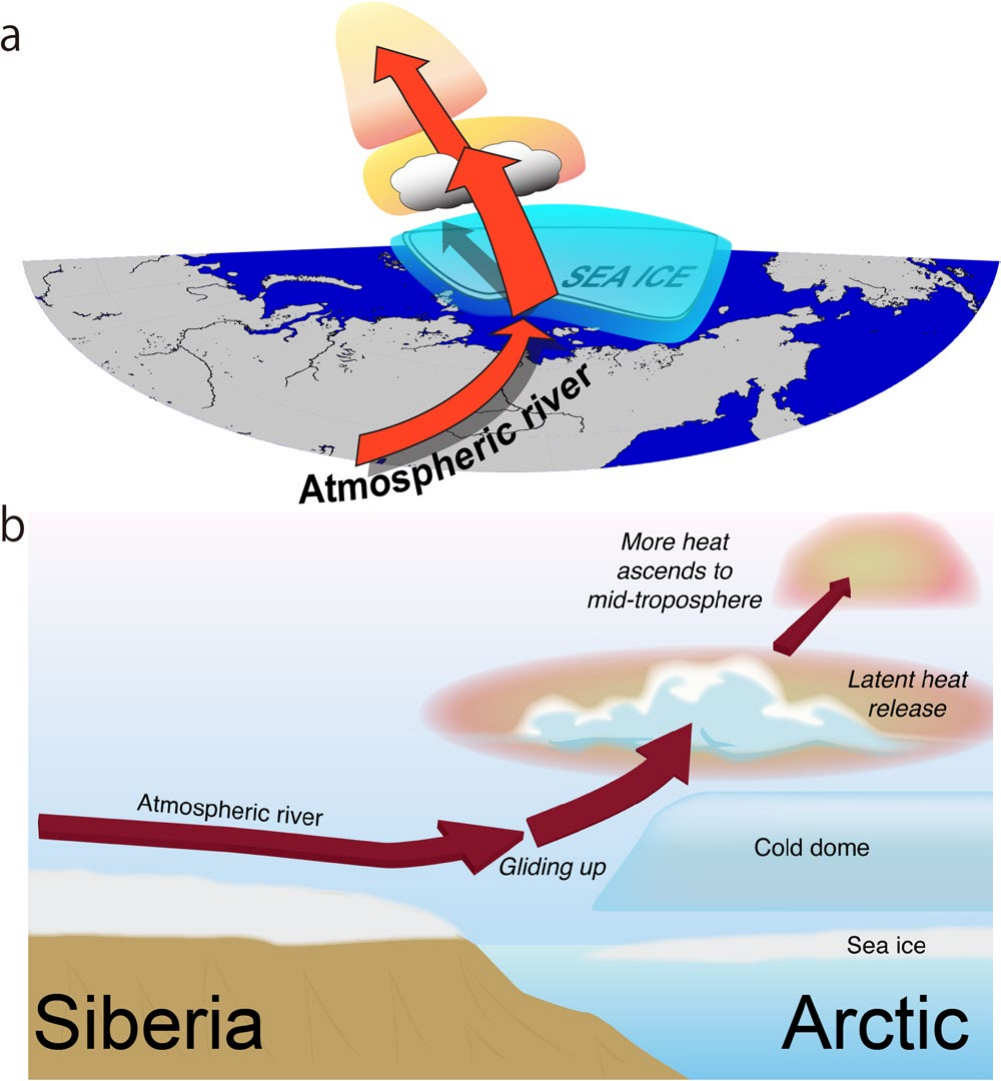
Schematics of tropospheric Arctic heating through the upward glide of Siberian atmospheric river. (**a**) Atmospheric rivers are river-style moisture flows from Siberia into the Arctic. The river-like glide upward and poleward over the cold air dome overlying the sea ice is shown. (**b**) Same as (**a**) but for viewed from the side. The Generic Mapping Tools (GMT) with version 4.5.6 (http://gmt.soest.hawaii.edu) was used to generate the map in this figure.

We here propose an hypothesis about heating mechanisms that potentially contribute to the warming of the Arctic mid-troposphere during the summer viewing from a land-ocean-atmosphere interaction system (Fig. 1). The hypothesis is based on radiosonde observations conducted during an expedition in August 2013 from the Russian icebreaker “Akademik Fedorov”. We document a new process, referred to as “Siberian atmospheric rivers”, which injects moist, warm air from Siberia over the ice-free Arctic Ocean in association with cyclonic weather systems. Because the surface air temperature above sea ice tends to be fixed at about 0 °C, a cool boundary layer capped by a strong temperature inversion forms in the lower troposphere over sea ice. This cold air near the surface maintains a meridional temperature gradient around the sea ice, and it blocks injection of Siberian atmospheric rivers into the cold boundary layer. This blockage of the boundary layer forces the Siberian atmospheric rivers to upglide. Poleward upglides of Siberian atmospheric rivers over the cold air dome overlying sea ice produce clouds that provide the upper atmosphere with heat via condensation of water vapour. This heating leads to increased buoyancy and further promotes ascent and heating of the mid-troposphere. These processes result in a coupling between the upper and lower atmosphere mediated by moisture injection from Siberian atmospheric rivers in interactions with local sea ice over the Arctic.

The present study focused mainly on documenting the existence of this process based on observations made from an icebreaker. First, we present observational signatures of an injection of warm, humid air over a near-surface cold air above the sea ice. Next, we show that a numerical simulation reproduces the phenomenon of Siberian atmospheric rivers gliding upward over the cold dome. We also conducted a numerical experiment with switching off latent heat associated with cloud formation, an experiment without Arctic sea ice, and an experiment with a weakened Siberian atmospheric river to test the impact of different components of the model on the robustness of the mechanism. The experiment with switching off latent heat can evaluate that the condensation of water vapour promotes an additional ascending and heating upper atmosphere. The experiment without sea ice can serve as an evaluation of the impact of near-surface cold air domes on the behaviour of Siberian atmospheric rivers. The experiment with a weakened Siberian atmospheric river can reveal the contribution of water vapour over land in Siberia to the Arctic mid-tropospheric temperature through the injection of the Siberian atmospheric river. Additional statistical analyses with a reanalysis dataset were used to document the historic existence of this process. We further compared the polar cap temperature change associated with Siberian atmospheric rivers between recent and past eras, but a quantitative assessment of the historic contribution of the proposed mechanism to Arctic warming is not a part of this paper.

## Results

### Observational results

**Figure 2:**
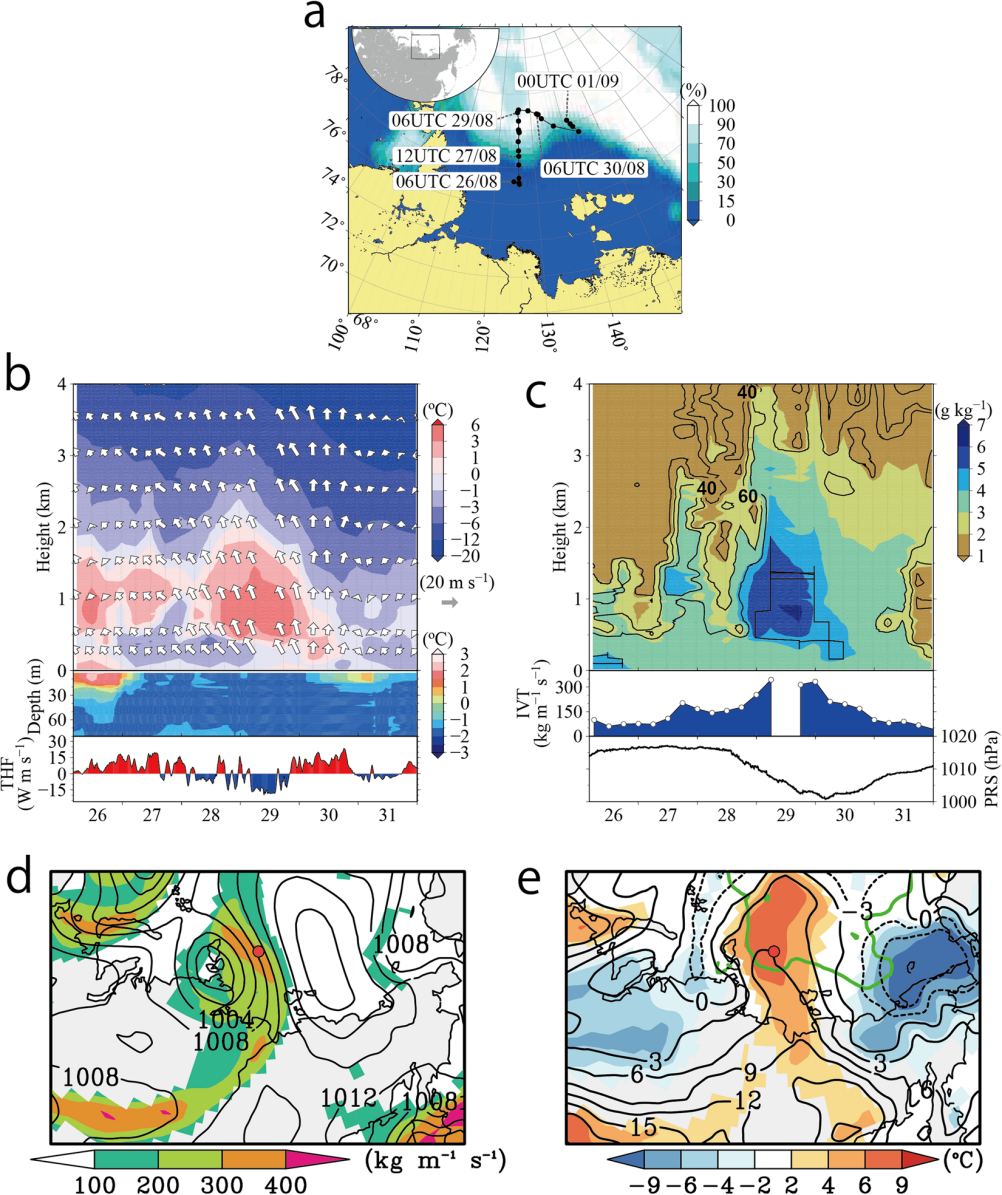
Map of the observational area and the atmospheric-oceanic vertical time section during the whole observation period, and synoptic scale atmospheric conditions at 06 UTC on 29 August. (**a**) Map of the observational area with time-averaged sea ice concentration (colour, %) and sounding points (circles) during the radiosonde observational period. (**b**) The atmospheric height-time section indicates temperature (°C) and horizontal wind vectors (m s^−1^). The oceanic depth-time section depicted ocean temperature (°C) and the upward turbulent sensible and latent heat fluxes from the surface (W m^−2^ s^−1^) are shown middle and bottom panel, respectively. (**c**) The top panel is same as (**b**) but for specific humidity (colour, g kg^−1^) and relative humidity (contours, %). The contour interval is 20%. The integrated water vapour transport (IVT) in the atmosphere was estimated to be between 1000 hPa and 300 hPa (kg m^−1^ s^−1^) and is presented in the bottom panel of the atmospheric plot. The surface pressure is also shown in bottom panel. (**d**) The map of sea level pressure (contour) and IVT (colour). Colours are masked over 100 kg m^−1^ s^−1^. The contour interval is 4 hPa. Red circle indicates the sounding point at 06 UTC on 29 August. (**e**) Same as (**d**) but for the temperature at 900 hPa (contours, °C) and the anomaly from the climatological values (colour, °C). The contour interval is 3 °C. The time-averaged ice edge defined by 50% sea ice concentration for the observation period is depicted using a green line. (**d**) and (**e**) use ERA-Interim. The Generic Mapping Tools (GMT) with version 4.5.6 (http://gmt.soest.hawaii.edu) and the Grid Analysis and Display System (GrADS) with version 2.1.a3 (http://cola.gmu.edu/grads) were used to generate the map in this figure.

We carried out 6-hourly global positioning system radiosonde measurements from 06 Coordinated Universal Time (UTC) on 26 August to 00 UTC on 01 September, during which time we observed Arctic sea ice from the ice-free ocean in the Laptev Sea (Fig. 2a). Figure 2b and c show atmosphere-ocean vertical sections recorded during our observations. The cross section in the period 26–29 August corresponds to a vertical-latitudinal section from the ice-free ocean to the sea ice zone transecting the ice edge. The change of air temperature near the surface during 26–29 August generally matched well with that of the underlying ocean: the air temperatures below an altitude of approximately 0.5 km above the sea ice were low, whereas the temperatures at the same altitudes over the ice-free ocean were relatively high (Fig. 2b). The temperature of the cold surface air over the sea ice stayed nearly constant, and a pronounced temperature inversion formed above the cold air under high surface pressure conditions until 12 UTC on 28 August. At the same time, high relative humidity was capped at the height of the temperature inversion, except for the night of 27 August (Fig. 2c). These thermal structures were similar to the dominant thermal structure in the Arctic documented by previous expeditions^30–32^: namely, the formation of a well mixed boundary layer with high relative humidity up to a height of 500 m. This well-mixed boundary layer is formed by a process that involves transformation of air masses. Warm air masses intruding from the continent to the sea-ice region are cooled by the release of heat to melt sea ice, and they are transformed into a shallow, well-mixed boundary layer capped by a temperature inversion^33^.

After 12 UTC on 28 August, very warm, humid air was observed centred at a height of approximately 1 km, and on 29 August the surface pressure was decreasing. This warm, humid air, which extended to an altitude of approximately 4 km, matched well with the increase in the southerly wind. In contrast, the air temperature near the surface over the sea ice did not rise continuously with downward turbulent heat fluxes. As a result, a strong vertical temperature inversion layer formed. The cloud top height defined by high relative humidity, however, penetrated beyond the temperature inversion layer (Fig. 2c and Supplemental Fig. 1a). The vertically integrated horizontal water vapour transport (hereafter called the integrated water vapour transport [IVT]; Methods) increased from 12 UTC on 28 August, with the maximum value reaching 344 kg m^−1^ s^−1^ at 06 UTC on 29 August (Fig. 2c). This value was more than triple the IVTs on 26 and 31 August.

Synoptic atmospheric conditions for the same time period (Fig. 2d) were determined using the gridded reanalysis product dataset ERA-Interim^34^. The reproducibility of the reanalysis was confirmed via comparisons with observations (see Supplementary Information 1). Cyclonic and anticyclonic systems were located on the western and eastern sides of the sounding point. An elongated IVT with a maximum greater than 200 kg m^−1^ s^−1^ extended from central Siberia; it covered a large area and was shaped like an atmospheric river. An IVT is much stronger in this state than in its basic state at high latitudes, but its strength is less than the threshold used to determine atmospheric rivers in mid-latitudes^35,36^ because the base amount of water vapour at high latitudes is generally less than that at mid-latitudes. At the same time, a warm region, corresponding to a large IVT, extended from central Siberia to the centre of the Arctic (Fig. 2e). The injection of moist, warm air was therefore generated by the northward progression of an IVT resembling a river and trailed by a cyclonic weather system. A recent paper has shown that atmospheric rivers are maintained by convergence within them and local evaporation around a cyclone^37^. At the same time, an IVT resembling a river does not always mean that the water is transported from far away along the IVT^38^. To examine the origin of the air masses associated with the moist, warm injection, we carried out a numerical experiment using *Lagrangian* trajectory analysis of air parcels.

### Numerical simulation and trajectory analysis

**Figure 3:**
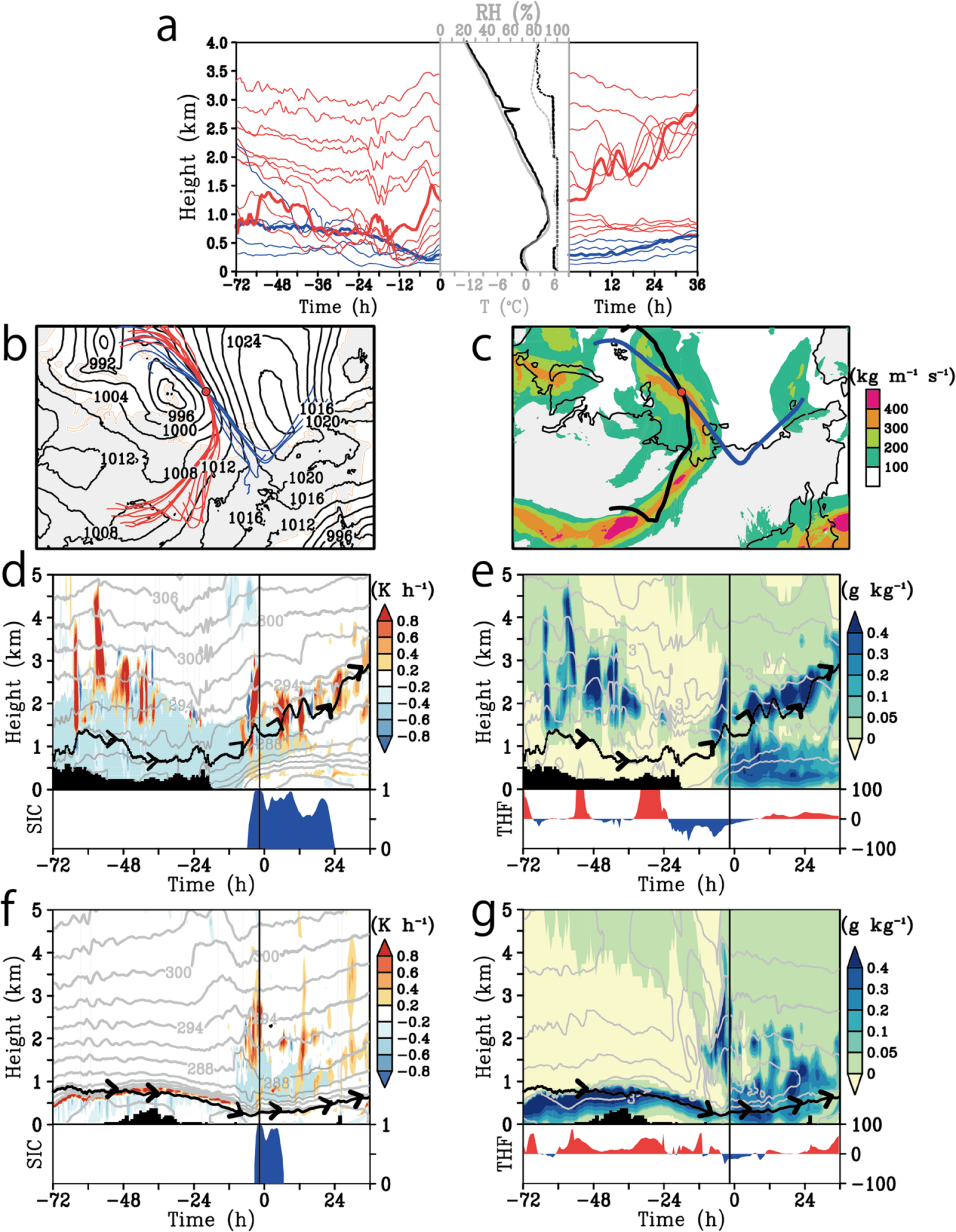
Model simulated backward and forward trajectories of air parcels initiated over the sounding point and time vertical section of the air trajectory along the Siberian route and Chukchi Sea route. (**a**) Time series of vertical displacements of air parcels over previous (to the sounding point) 72 hours and next 36 hours (after the sounding point). The Siberian route and the Chukchi Sea route are shown as red and blue lines respectively. Bold line indicates the air parcels initiated 1.2 and 0.3 km height. The middle panel indicates the observed (black) and simulated (grey) vertical profiles of temperature (solid line, °C) and relative humidity (dotted line, %) over the sounding point. (**b**) The map of sea level pressures (contours, hPa) at the starting time of the trajectories. The contour interval is 4 hPa. The red and blue lines indicate the horizontal pathways of the Siberian route and Chukchi Sea route, respectively. The red circle indicates the location of sounding. (**c**) Same as (**b**) but for IVT values. The black and blue lines indicate horizontal pathways of the trajectories initiated at 1.2 km and 0.3 km, respectively. (**d**) Time vertical section along the black line in Fig. 3c in terms of its potential temperature (grey contours, K), condensation heating rate (colour, K h^−1^), and specific humidity over 5 g kg^−1^ (blue mark). The contour interval is 3 K. The black lines indicate the trajectories of air parcels, and the black mask shows the topography. The bottom panel indicates the sea ice concentration (10^2^%). (**e**) Same as (**d**) but for the cloud water and ice mixing ratio (colour, g kg^−1^) and specific humidity (contour, g kg^−1^). The contour interval is 1 g kg^−1^. The bottom panel indicates the turbulent sensible and latent heat fluxes from the surface (W m^−2^). (**f** and **g**) are same as (**d** and **e**) but along the blue line in Fig. 3c. The Grid Analysis and Display System (GrADS) with version 2.1.a3 (http://cola.gmu.edu/grads) was used to generate the map in this figure.

We executed a numerical simulation to reproduce the cyclonic episode captured by *in situ* observation (the design of simulation is in Methods). The simulated values are almost identical to those observed (Supplementary Information 1). The trajectories of the air parcels above a sounding point over the sea ice showed two pathways; one from Siberia at altitudes above 0.5 km, and the other from the Chukchi Sea at altitudes below 0.5 km (Fig. 3a and b). The vertical structure observed at 06 UTC on 29 August apparently showed the warm air intrusion from continental side by south-easterly wind, but the upper warm and lower cold air masses had quit different origins.

The Siberian route is overlapped by the large IVT area (Fig. 3c). This large IVT area could be an atmospheric river transporting water from central Siberia to the Arctic. The air parcels travelling along this route generally followed higher routes over the 12 hour period, and continued rising to further higher levels expect that lower several parcels trapped in the temperature inversion layer (Fig. 3a). The vertical time sections along the trajectory of an air parcel originating at 1.2 km above the sounding point (Fig. 3d,e and black line in Fig. 3c) show the movement of high specific humidity, i.e., over 5 g kg^−1^, from land to the Arctic at altitudes below 2 km, and its upward glide over the cold air (light blue shade in Fig. 3d). The upward and poleward upgliding of the humid air parcels further promoted the formation of additional upper–level stratus clouds above the low-level clouds (Fig. 3e), and they released latent heat into the atmosphere through the condensation of water vapour (Fig. 3d). The air parcels, forced to warm via heat release due to condensation, can travel to even higher levels across isentropic lines. This upward slantwise motion after upgliding over surface cold air is similar to warm air motion over a warm front associated with the atmospheric river.

In contrast, the air parcels from the Chukchi Sea was confined in the cold air mass and they continued traveling at the same levels after passing the sounding point as before (Fig. 3a). The origin of the clockwise Chukchi Sea route was an anomalous cold air region associated with an anticyclonic system over the sea ice on East Siberian Sea (Fig. 2e). The tracking of an air parcel initiated at 0.3 km (Fig. 3f,g and blue line in Fig. 3c) shows that the air parcel was trapped by the strong temperature inversion layer and crawled to the sounding point forming the low-level clouds (Fig. 3f,g). The vertical thermal structures along this trajectory were similar to the dominant thermal structure in the Arctic^32^, thus the source of surface cold air was over the sea ice rather than over the continent. The isentropic lines below 1 km were gradually descending toward the sounding point with time, indicating that the height of the cold air mass was shrinking via heating from the land and ocean surface and warm air advection. The declining of isentrope was, however, inhibited over the sea ice, thus the cold air masses were kept cold by the sea-ice cooling. These results imply that a cold air dome is associated with sea ice located over the Arctic Ocean.

**Figure 4:**
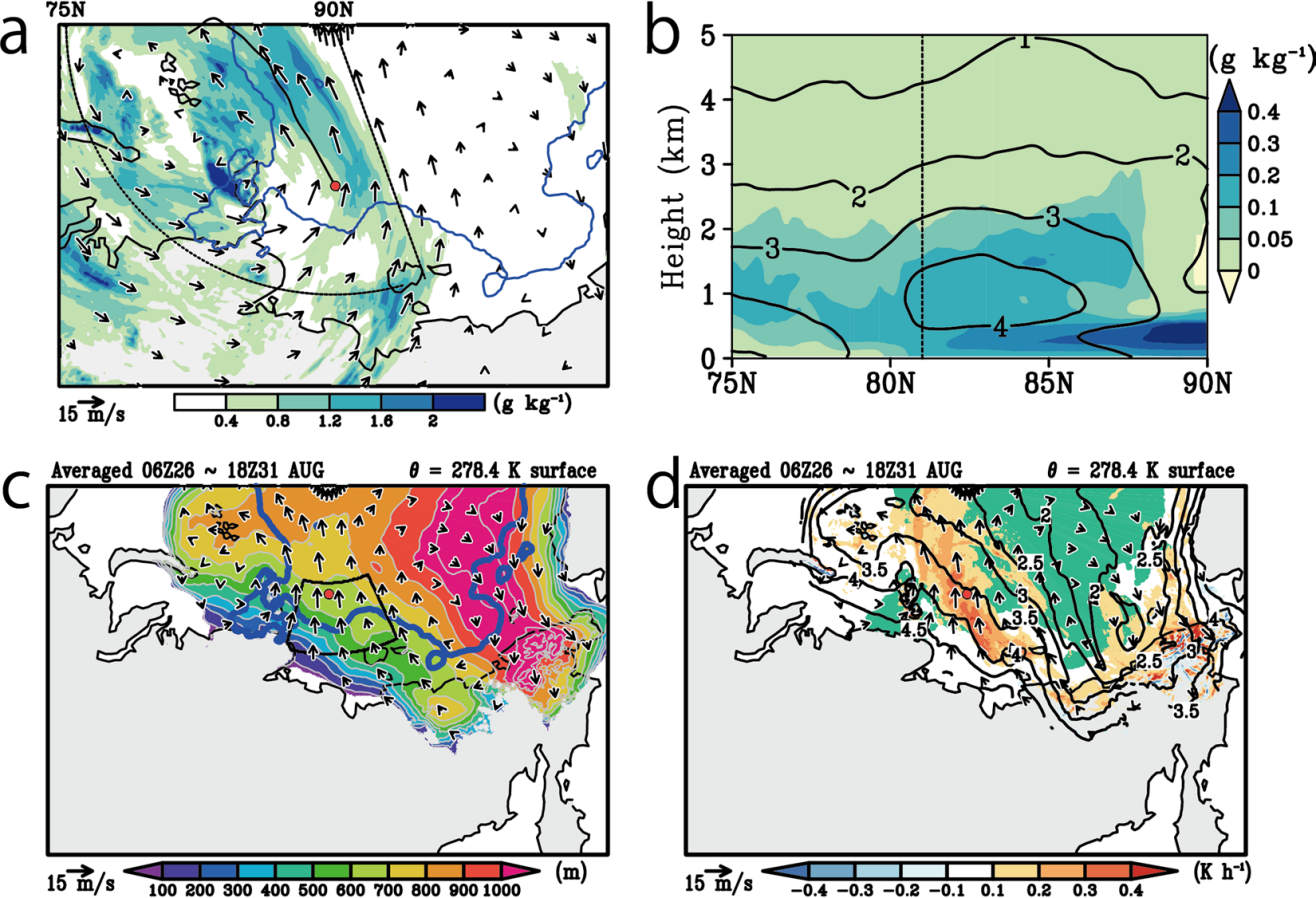
Model simulated map of the 24-hour mean cloud water and ice mixing ratio with horizontal wind and those of the latitude-vertical section averaged from 35° E to 140° E, and the averaged map of cold air dome characterized by the isentropic surface of 278.4 K. (**a**) The temporary averaged map of the vertically integrated cloud water and ice mixing ratio (colour, g kg^−1^) and vertically averaged horizontal wind (vector, m s^−1^). The vertical integrating and averaging are from 1.2 km to 4 km, and the temporal averaging period are from 08 UTC on 29 to 08 UTC on 30 August. The blue lines indicate the sea ice concentration of 10%. A large sector with dotted lines is the region for loditudilanl averaging showing in Fig. 4b. (**b**) Temporary averaged latitude-vertical section averaged from 35° E to 140° E in terms of cloud water and ice mixing ratio (colour, g kg^−1^) and specific humidity (contours, g kg^−1^). The temporal averaging period is the same as (**a**). The contour interval is 1 g kg^−1^. The black dots line indicates the latitude of sounding at 06 UTC on 29 August. (**c**) The map of height (colour and contours, m) and horizontal wind (vector, m s^−1^) along the simulated isentropic surface of 278.4 K averaged from 06 UTC on 26 to 18 UTC on 31 August. The contour interval is 100 m. The blue line indicates the sea ice concentration of 10%, and the black line shows the defined area of the Laptev Sea. (**d**) Same as (**c**) but for the condensation heating (colour, K h^−1^) and specific humidity (contours, g kg^−1^). The contour interval is 0.5 g kg^−1^. The green mask indicates the sea ice defined by the sea ice concentration over 10%. The red circle in (**a**,**c** and **e**) indicates the location of sounding at 06 UTC on 29 August. The Grid Analysis and Display System (GrADS) with version 2.1.a3 (http://cola.gmu.edu/grads) was used to generate the map in this figure.

In addition to these *lagrangian snapshot-like* views, we show time-averaged ones (Fig. 4). The upglide signature seen by cloud water and ice mixing ratio is also detected in time-averaged fields; clouds between 1.2 km and 4 km were extended to the higher latitudes along with the strong south-easterly wind from around sounding point (Fig. 4a) and this structure can be seen in zonally averaged vertical section (Fig. 4b). Dome-like structure of surface cold air mass characterized by an isentropic surface at 278.4 K are also seen in Fig. 4c (definition of the cold air mass are in Method). Cold air covered not only within the sea-ice region but also spreaded over the whole Arctic Ocean, and strong meridional gradient of the height of the isentropic surface was around the land-ocean boundary. Moreover, we can identify the shape of the cold air as a convex dome centred over the north of the Chukchi Sea, and its peak height reached over 1 km. Over the Laptev Sea, the southerly wind blew along the slope of the cold dome with gaining its altitude, where the release of the condensation heat and the drop of the specific humidity are seen (Fig. 4c,d). Prominent condensation heating was also identified at the southern part of the observation points, corresponding with the ice edge rather than the land-ocean boundary. This implies that the warm-humid air mass coming from the outside of the Arctic frequently saturated aloft there with gliding along the slope of isentropes during the observation period because these features were seen by the time average, not by the temporary.

In summary, warm air from Siberia brought by a cyclonic system collided with the area of the cold air dome, which acted like a barrier, and the warm air was forced to glide up over the cold dome. This structure appears to be similar to a mid-latitude synoptic-scale frontal system. Although the structure appears similar, the contribution from the surface conditions is different: The mid-latitude frontal systems do not necessary develop because of surface conditions such as SST gradient, while, in our case, the surface cooling condition was essential.

### Sensitivity experiment

Next, we executed numerical experiments with switching-off the latent heat release (DRY), the ice-free ocean surface (NoICE) and reduced Siberian humidity runs (RHcut). In DRY, we switched off the latent heat release after 08 UTC on 29 August, at which forward trajectory calculation stated. In NoICE, the SST, which is a surface boundary condition, was fixed at 4 °C in the whole Arctic Ocean, and a preexisting cold dome was broken in order to suppress a cold dome development. In RHcut, we extremely reduced the water vapour above land in Siberia (see Methods). Comparisons of the aforementioned simulation (Control run: CTL) with DRY, NoICE, and RHcut further support the process illustrated in Fig. 1.

**Figure 5:**
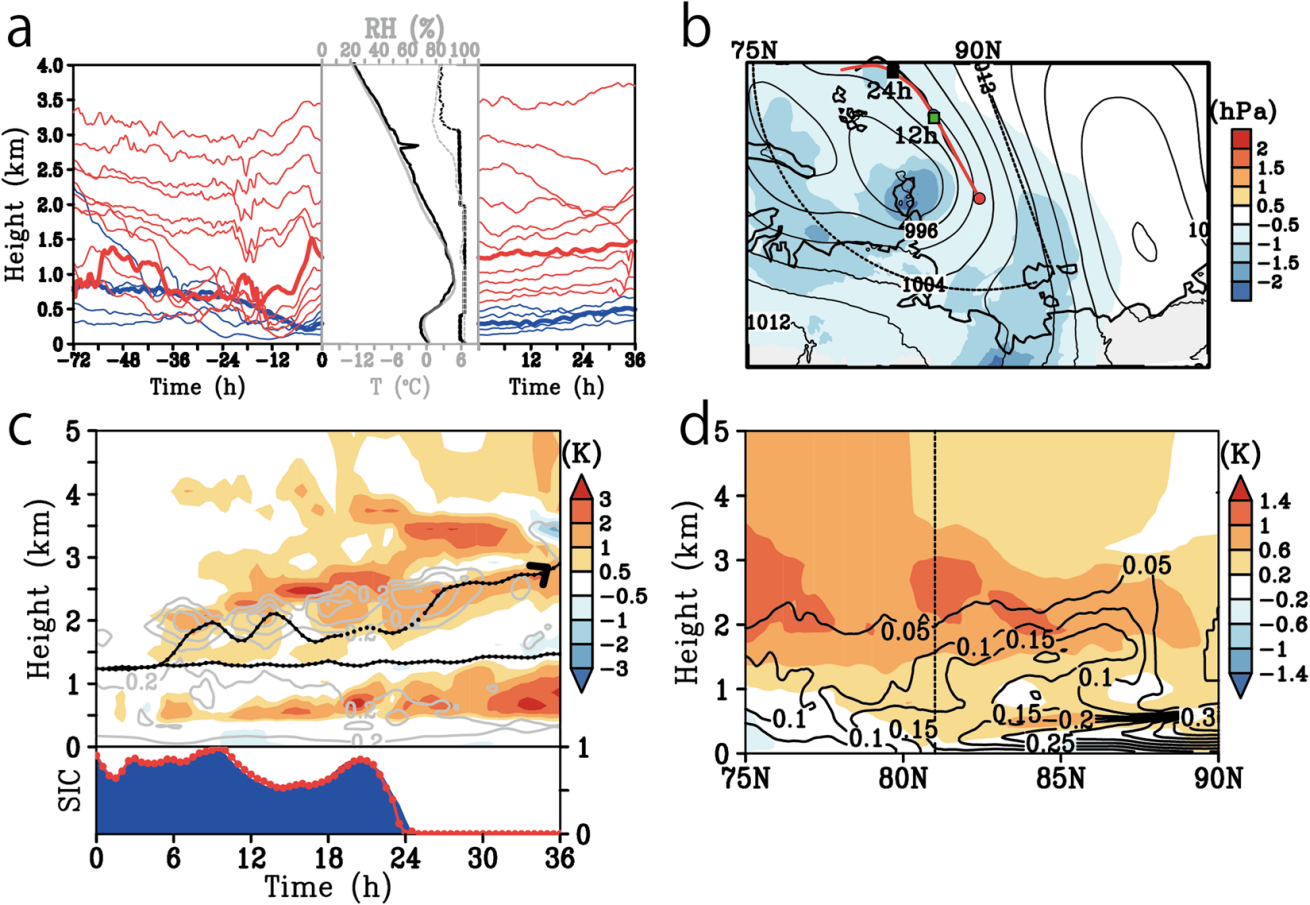
Model-simulated back and forward trajectories of the DRY, and the difference of the averaged sea level pressure and potential temperature fields between the CTL and DRY. (**a**) Same as Fig. 3a but for DRY. (**b**) Temporally averaged sea level pressure in CTL (contour, hPa) and differences from those of DRY (colour, hPa). Negative value indicates that CTL is lower than DRY. The temporal averaging period is from 08 UTC on 29 to 08 UTC on 30 August. The contour interval is 2 hPa. The red circle indicates the location of sounding at 06 UTC on 29 August. Black and red line shows the horizontal pathways of forward trajectories of the CTL and DRY respectively. Green and black squares indicate the location of air parcels at 12 and 24 hours later from trajectory started time. A large sector with dotted lines is the region for longitudinal averaging shown in Fig. 5d. (**c**) Time vertical section along the forward trajectories initiated 1.2 km in terms of its difference of potential temperature between CTL and DRY (colour, K) and cloud water and ice mixing ratio in CTL (grey contours, K). Positive value of the difference indicates that CTL is warmer than DRY. The contour interval is 0.2 g kg^−1^. Black dots lines show the trajectories of air parcels in CTL and Dry respectively. The bottom panel indicates the sea ice concentration in CTL (blue mask, 10^2^%) and DRY (red line, 10^2^%). (**d**) Same as Fig. 4b but for the difference of potential temperature between CTL and DRY (colour, K) and cloud water and ice mixing ratio in CTL (contour, g kg^−1^). Positive value of the difference indicates that CTL is warmer than DRY. The Grid Analysis and Display System (GrADS) with version 2.1.a3 (http://cola.gmu.edu/grads) was used to generate the map in this figure.

The DRY demonstrates the contribution of latent heat to the upglide and heating the Arctic upper air. No prominent upglides as in CTL were seen in the trajectories of DRY (Fig. 5a). Thus the thermal forcing associated with condensation dominantly activated the upglide rather than dynamical forcing such as a large-scale vertical motion. Moreover, a cyclone system in CTL was stronger than in DRY (Fig. 5b). Time vertical section following the air parcel (Fig. 5c) signifies that the rising of an air parcel in CTL started with releasing the latent heat and forming the cloud after 6 h, and the temperature anomaly from DRY reached about 1 K in the cloud. After that, vertically stepwise temperature anomalies developed parallel to the top of cloud forming, and the temperature anomaly maxima reached over 3 K. Positive temperature anomalies were also seen between 0.5 and 1 km height, which could contributed to the strengthening of a temperature inversion layer. The strengthened inversion layer trapped the lower air parcels inside its layer and tended to divide the lower and upper air masses (see Figs 3a and 5a). These thermal structure forced by condensation was also seen by the zonally averaged latitudinal-vertical section (Fig. 5d). The peak of positive anomalies is located between 2 and 3 km height in higher latitudes along with those of lower latitudes. The peaks in higher latitudes correspond with the top of a cloud layer, which is similar structure to those along an air trajectory showing in Fig. 5c.

**Figure 6:**
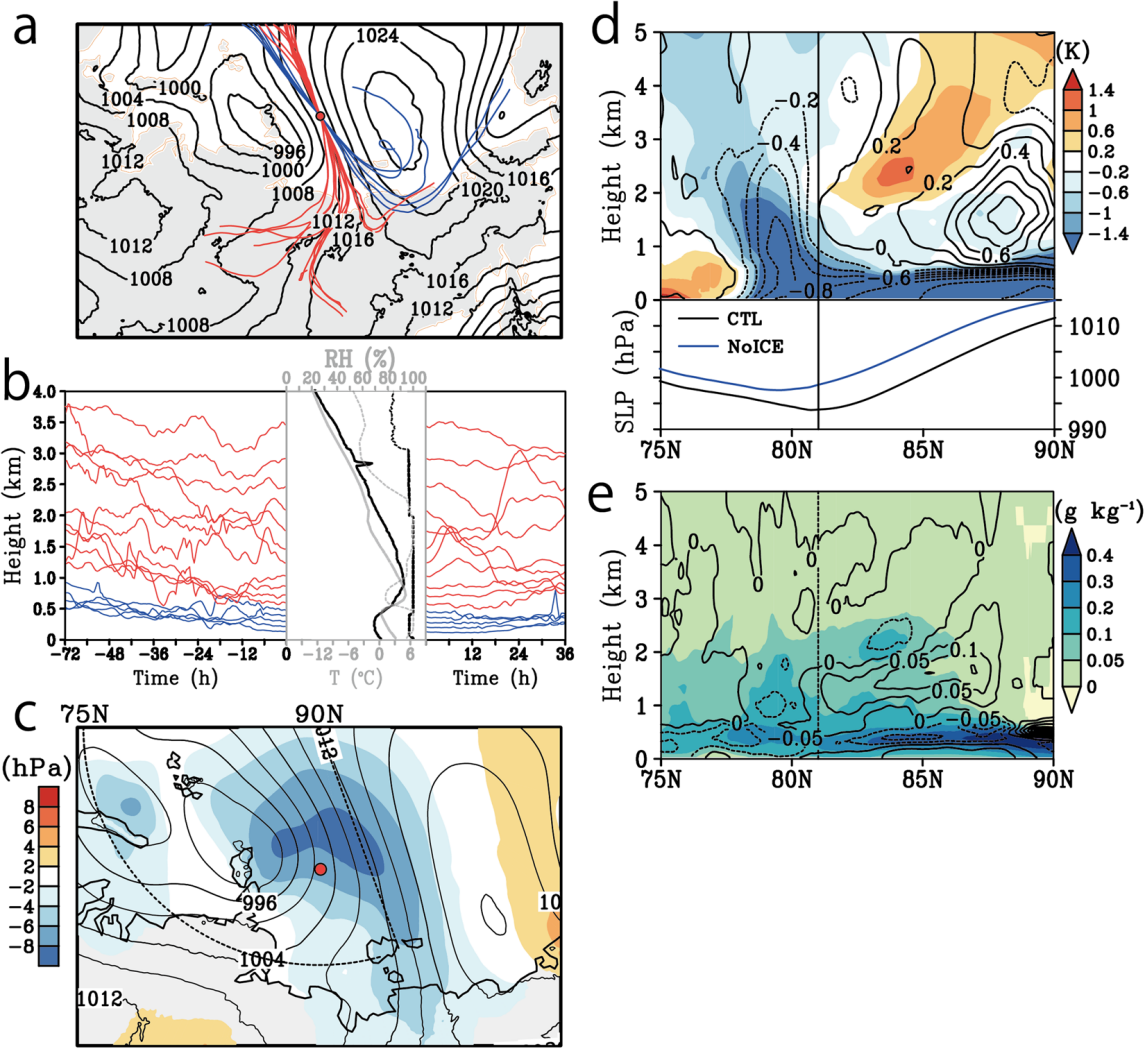
Model-simulated back and forward trajectories of the NoICE, and the difference of the averaged sea level pressure, potential temperature, and water vapour fields between the CTL and NoICE. (**a**) and (**b**), same as Fig. 3a and b, respectively, but for NoICE. (**c**) Same as Fig. 5b but for the differences between CTL and NoICE. The negative values indicate that CTL is lower than NoICE. (**d**) Top panel is same as Fig. 4b but for the difference of potential temperature (colour, K) and specific humidity (contours, g kg^−1^) between CTL and those of NoICE. Positive value indicates that CTL is warmer and wetter than NoICE. Bottom panel shows the sea level pressure in CTL and NoICE respectively. (**e**) Same as Fig. 4b but for the cloud water and ice mixing ratio in NoICE (colour, g kg^−1^) and the difference between those of CTL and NoICE (contour, g kg^−1^). Positive value indicates that CTL is cloudier than NoICE. The Grid Analysis and Display System (GrADS) with version 2.1.a3 (http://cola.gmu.edu/grads) was used to generate the maps in this figure.

The differences between the NoICE and CTL demonstrate the role of sea ice in this process. The vertical displacements of trajectories in the NoICE differed from or were in the opposite direction to those of CTL (Fig. 6a and b): the air parcels below 1 km travelled at constant levels from approximately 12 hours before the observational point of the sounding and 36 hours after without rising, also the upper air parcels moved downward with time rather than upward. The atmosphere above 1 km in the NoICE was simulated as more likely to be cold and dry than that of CTL (middle panel in Fig. 6b). In addition, the weakening of the cold dome can lead to weaker baroclinicity over the Arctic Ocean thus the cyclonic system in NoICE could not develop as strong as in CTL (Fig. 6c and bottom panel in 6d). The trough of sea level pressure in CTL was deeper than NoICE over the Laptev Sea, thus intensified zonal pressure gradient strengthens poleward wind, which encourages the transportation of the warm and humid air mass to higher latitudes (Fig. 6c). The centre of the cyclonic system in CTL also located more northern than NoICE (Fig. 6d). The latitudinal vertical section of anomalies of potential temperature and specific humidity indicate that the CTL was warmer and wetter than NoICE in upper levels in high latitudes (Fig. 6d). Above 2 km height, positive and negative temperature anomalies in CTL were seen in the northern and southern parts of the cyclonic system respectively. This northern warm and southern cold pair is apparently corresponded with the intensification of the cyclonic system by the existence of the cold air dome. In addition, cloud associated upglide of air parcel was less in NoICE than CTL, and positive anomalies of cloud water and ice mixing ratio in CTL was seen at the bottom of the positive temperature anomalies (Fig. 6d and e). This implies that weakening of the cold air dome damps the cyclonic system, and inhibits the intrusion and upglide of Siberian atmospheric river to the poleward side.

**Figure 7:**
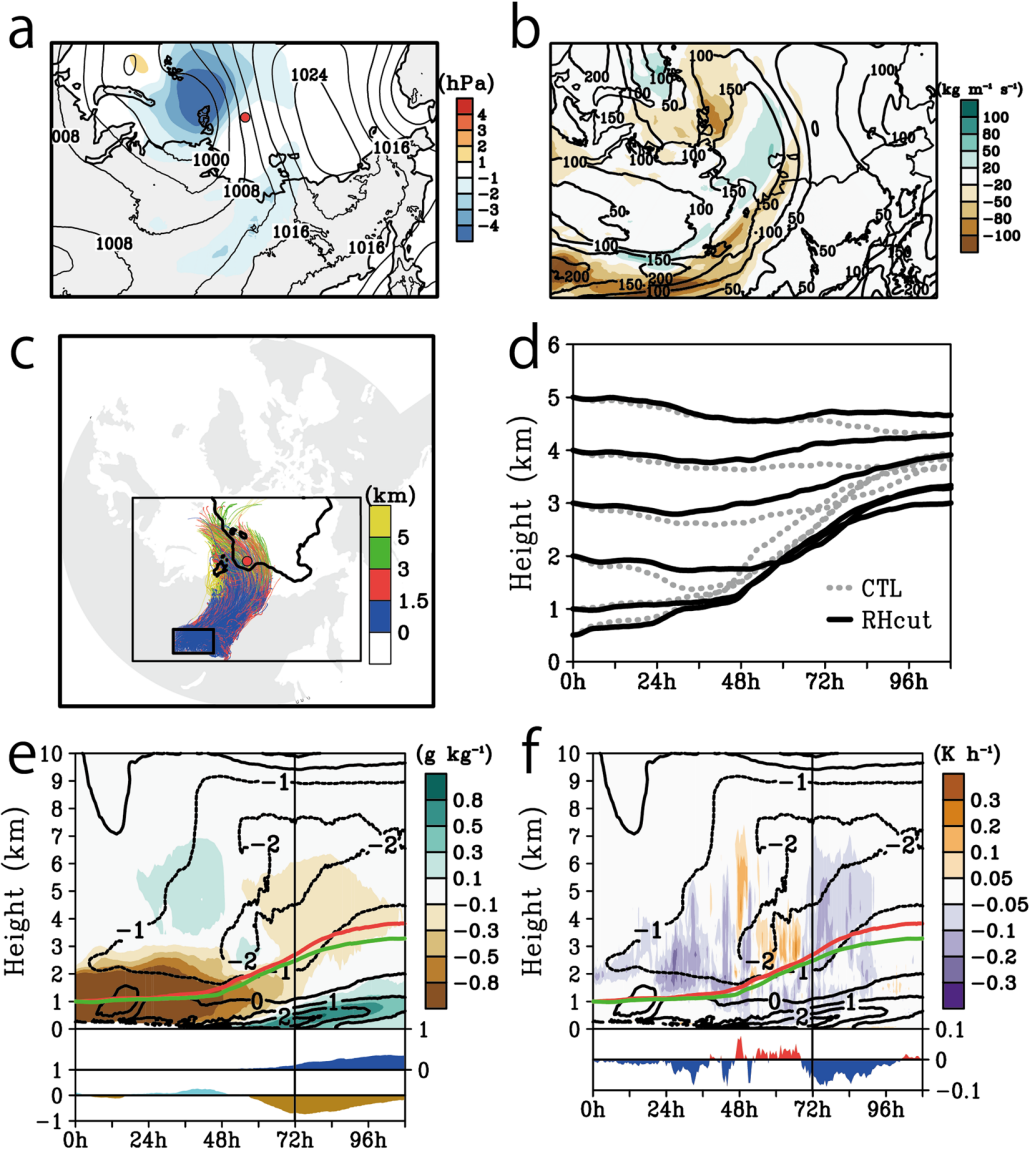
Model-simulated synoptic atmospheric conditions, forward trajectories initiated around central Siberia and the difference in the averaged vertical time section along the trajectories between the CTL and RHcut. (**a**) Same as Fig. 5b but for the differences between CTL and RHcut (colour, hPa) averaged from 08 UTC on 28 to 20 UTC on 30 August. The negative values indicate that CTL is lower than RHcut. (**b**) Map of IVT in the RH (contour) and the difference between those in CTL and RHcut averaged in same period of (**a**). Negative values indicate that the RHcut is drier than the CTL. The contour interval is 50 kg m^−1^ s^−1^ (**c**) Horizontal pathway of the 108 hour projections in the RHcut for five hundred air parcels originating at an altitude of 1 km in the small black square. Colour indicates the height of each parcel. (**d**) The composite vertical displacements of the trajectories of the parcels released at 0.5, 1, 2, 3, 4, and 5 km in the CTL (dashed line) and RHcut (solid line). (**e**) Difference in the specific humidity (colour) and temperature (contour) between RHcut and CTL. Negative values indicate that the RHcut is drier and cooler than the CTL. The panel represents a composite Lagrangian vertical time section along the forward trajectory of a parcel released at 1 km. The sea ice concentration in the CTL and the differences of the precipitable waters from 750 hPa to 300 hPa in the RHcut from those in the CTL (bottom panel) are shown in the middle and bottom panels, respectively. (**f**) Same as (**e**) but for condensation heating scenarios (colour, K h^−1^) and the difference of the vertically integrated condensation heating from 750 hPa to 300 hPa is shown in the bottom panel. Negative values indicate that the RHcut has less heat than the CTL. The red and green lines in (**e**) and (**f**) show the composite vertical displacement of the air parcels of CTL and RHcut, respectively. The Grid Analysis and Display System (GrADS) with version 2.1.a3 (http://cola.gmu.edu/grads) was used to generate the map in this figure.

The reduction in water vapour above Siberia resulted in two effects: the weakening of the Atmospheric Rivers and the cyclonic system (Fig. 7a and b). The map of time averaged IVT in RHcut resembled a river, but the penetration of the Atmospheric river into the high latitudes was reduced (Fig. 7b). The horizontal pathways of air parcels released at 1 km over Siberia in the RHcut showed that the air parcels travelled to the Arctic and rose a level at the ice edge (Fig. 7c). The composite vertical displacements, which were derived from averaging trajectories released at each level are shown in Fig. 7d: the air parcels that originated below 2 km rose, reaching 4 km in the CTL, whereas in the RHcut, they rose less. We constructed a composite *Lagrangian* vertical time section along 500 trajectories initiated at 1 km over Siberia (see black square of Fig. 7c). The middle troposphere, between 3 km and 7 km above the sea ice, was drier and colder in RHcut than in CTL (the latter 60 h shown in Fig. 7e). Precipitable water, which is the vertically integrated specific humidity between 750 hPa to 300 hPa, decreased above the Arctic by up to −0.7 kg m^−2^ in the RHcut from that observed in CTL. The negative anomalies of the condensation heating also corresponded to the negative anomalies of temperature and specific humidity (Fig. 7f). In the latter 72 hours, the condensation heating in RHcut was inactive throughout the middle troposphere, with a vertical integral between 750 hPa and 300 hPa reduced by up to −0.12 K h^−1^ compared to CTL. These differences imply that the reduction of water vapour source above central Siberia can result in cooling of the middle troposphere of the Arctic.

This section tested the contribution of each component – latent heat, cold air dome associated with sea ice, and the water vapour over the Siberia – to the process illustrated in Fig. 1. All the elements contributed to the heating upper air over the Arctic through the intensification of a cyclonic system with poleward upgliding of Siberian atmospheric river. These results suggest that the interaction of land-ocean-atmosphere could amplify the Arctic upper air warming through the modification of a cyclonic system.

### Statistical analysis

The observational and numerical results imply that the water vapour was transported by a cyclonic system that injected moisture in a form that resembled a river from central Siberia toward the Arctic Ocean. To demonstrate that the injections have occurred in the past, we executed additional statistical analyses using a reanalysis product. We also evaluated the temperature rise over the Arctic during the recent era as compared with the past, when Siberian atmospheric river events occurred (details are in Methods).

**Figure 8:**
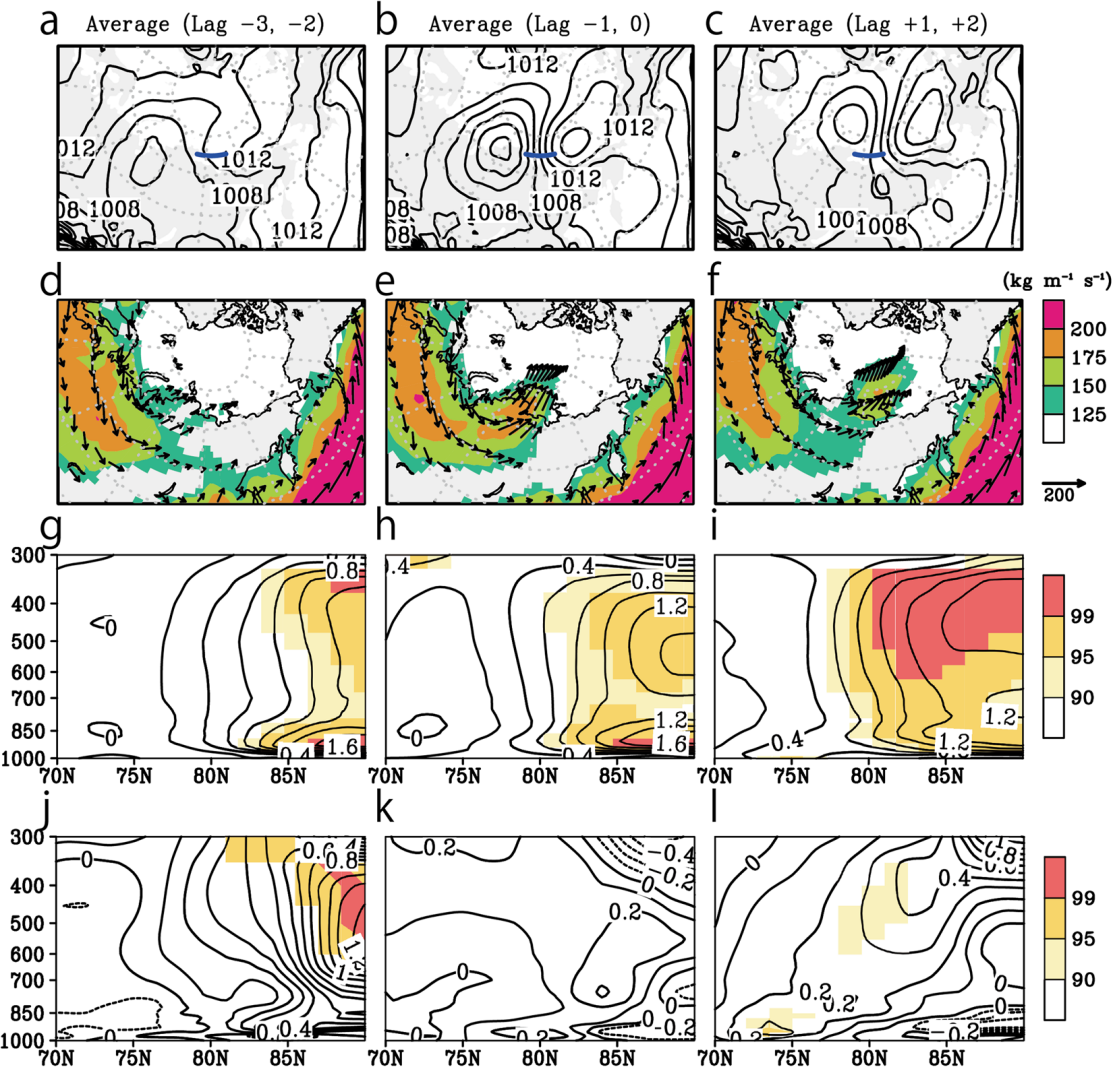
Lag composite map and, the anomaly of zonal mean polar cap temperature and that of tendencies between negative and positive ice year in the Laptev Sea associated with the Siberian atmospheric rivers events. (**a**) The composite map of sea level pressure (hPa) with 2 days lead from the day of the Siberian atmospheric rivers passed through the blue line at 75° N. The contour interval is 2 hPa. (**b**) same as (**a**) but for the day of Siberian atmospheric rivers passed the blue line. (**c**) same as (**a**) but for 2 days lag. (**d**,**e** and **f**) are same as (**a**,**b** and **c**) but for the IVT (colour in kg m^−1^ s^−1^) and vertical integrate moisture flux (vector). (**g**,**h** and **i**) are same as (**a**,**b** and **c**) but for the pressure-latitude section about the anomaly for zonal mean temperature (contour, K) between negative and positive ice year. The contour intervals is 0.2 K. The colour indicates the confidence level of the anomaly between negative and positive ice year. (**j**,**k** and **l**) are same as (**g**,**h** and **i**) but for the temperature tendency (contours, K 2 day^−1^). The Grid Analysis and Display System (GrADS) with version 2.1.a3 (http://cola.gmu.edu/grads) was used to generate the map in this figure.

Figure 8a–f show the transition of daily composite maps of sea level pressure, IVT, and vertically integrated moisture flux during the time when an anomalous northward meridional moisture flux crossed the coast of the Laptev Sea. We found 147 days with Siberian atmospheric river episodes during the summer (June, July, and August) between 1979 and 2013, an indication that intense moisture intrusions occur at least once per month. When an anomalous moisture intrusion occurs, a cyclonic system tends to move from the northern coast of central Siberia to the centre of the Arctic Ocean and to pass on the west side of the Laptev Sea (Fig. 8a–c). A pair of cyclonic and anticyclonic systems is also apparent, a pattern quite similar to our observations (Figs 2d and 8b). Eastward moisture flux and a large IVT area are always apparent on the west side of Siberia (Fig. 8d–f). Along with the movement of the cyclonic system, the large IVT area expanded to the Arctic Ocean from central Siberia and formed a flow resembling a river directed toward the central Arctic (Fig. 8e,f). These features look very similar to the features that we observed. The observational and numerical results thus depicted a typical case of Siberian atmospheric rivers.

We next evaluated the sensitivity of the Siberian atmospheric rivers in situations when the sea ice decreased (Methods). We divided the members of the composite into two groups associated with the inter-annual variability of the summer sea-ice concentration in the coastal area of the Laptev Sea (the region was 75–80°N, 100–140°E); one group was taken from the years of positive ice anomalies, and the other group was taken from the years of negative ice anomalies. This separation corresponded closely to the distinction between past and recent years. We averaged the zonal mean temperature over the Arctic in positive and negative years with eliminated the long-term linear trend. Figure 8g–i show the anomaly of zonal mean temperature in negative years compared to positive years during Siberian atmospheric rivers episodes. The recent warming signals were retained, but the positive temperature anomaly in the troposphere was increasing with time. This signal implies that the Arctic troposphere tended to be warmed more by the injection of Siberian atmospheric rivers in the years when the sea ice over the Laptev Sea was below normal. We also compared the temperature tendencies during Siberian atmospheric river episodes (Fig. 8j–l). The positive anomaly of temperature tendencies, corresponding to the growth of the positive temperature anomaly (Fig. 8i), tilted northward with height between 75°N and 85°N (Fig. 8l). A weak statistical significance was apparent in the lower troposphere above 75°N and in the middle troposphere above 80°N. This tilted warming pattern might reflect upglide heating by the Siberian atmospheric rivers because the front of the surface cold dome during negative ice years was located around 80°N. These results led us to conclude that the Siberian atmospheric rivers are potentially an important contributor to the recent Arctic tropospheric warming.

## Discussion and Conclusion

This paper documented the existence of Siberian atmospheric rivers on the basis of observational, numerical, and statistical analyses. Numerical simulations indicated that our observations captured one of the episodes of upglides of Siberian atmospheric rivers over the near-surface cold dome. The historical analysis suggests that this process is not unusual and in fact quite common. In addition to the upglide process, there is a possibility of an air mass transformation process^33^, which would be able to keep the boundary-layer air mass cool because of the underlying sea ice, even if the intruded warm air were partially entrained into the boundary layer. The cooling process by melting of sea ice^33^ enables the cold air dome to spread out the ice-free area in a ice-melting season, although the formation and maintenance processes of the cold air dome is beyond the scope of this study. The proposed concept of the upglides over the cold air dome has similar condition to the occurrence of mid-latitude diabatic Rossby waves^39,40^, in which the positive low-level potential vorticity anomaly induces a poleward low-level jet of warm moist air at its downstream side. This stream ascends along the poleward-sloping isentropes until condensation occurs^39^.

The sensitivity experiments revealed that both the presence of sea ice and a sufficient supply of moisture from Siberia are important factors in the formation of upglides of Siberian atmospheric rivers. In all the experiments, the cyclonic intensity was weaker than during the CTL. Both atmospheric rivers and sea ice strengthened the cyclonic system, and they strengthened the tropospheric heating by strengthening the cyclone system. These results imply that a poleward gradient of surface thermal conditions such as sea-ice concentrations has an influence on synoptic-scale cyclone activity. A similar influence of a surface thermal gradient on synoptic-scale cyclone activity has commonly been found over mid-latitude regions where there are large south-north SST gradients, such as the Kuroshio Current and Gulf Stream^41,42^. Because this sea-ice-induced thermal gradient over the Arctic is probably larger than that of the mid-latitude SST gradient, it is logical to assume that the sea-ice-induced cyclone developing mechanism, such as shown in the present study, is not unusual over the Arctic region.

Local evaporation and moisture convergence are important for the formation and maintenance of atmospheric rivers^36^. There could be a positive feedback link through which Siberian atmospheric rivers could intensify a cyclone and the associated mid-tropospheric poleward heat transport; the intensified cyclone could then further strengthen the atmospheric rivers. Recently, the water vapour and precipitable water over eastern Siberia have been increasing^25^, and a positive precipitation trend has also been identified with a change of atmospheric circulation to cyclonic over the northern Eurasia^26,27^. The increased humidity of the land surface due to the increase in summer precipitation because of cyclone activity also contributes to further warming and wetting in the active layer of the permafrost^28^. The proposed concept of Siberian atmospheric rivers might link changes in hydrological regimes in Siberia with warming of the Arctic mid-tropospheric temperature. Our statistical analysis (although the statistical significance was weak) implies that the recent Arctic mid-tropospheric temperature increase is likely due to warming caused by Siberian atmospheric rivers, but we cannot claim that the relationship is robust. Finally we emphasize that our proposed concept includes individual arctic warming mechanisms as a view from a land-ocean-atmosphere interactive system.

## Methods

### Radiosonde launches

6-hourly radiosonde measurements were carried out from 06 UTC, 26 August to 00 UTC, 01 September, with trajectory starting in the ice-free ocean and ending in the ice zone in the Laptev Sea. Radiosonde launches were conducted from a Russian icebreaker “*Akademik Fedorov”* as a part of the 2013 cruise of the Nansen and Amundsen Basins Observational System program.

To obtain a uniform vertical resolution of the radiosonde data, we averaged the data vertically over intervals of 20 m and then smoothed the data by the weighted running mean over intervals of 100 m to remove small perturbations. The ocean temperature was sampled by a conductivity temperature depth profiler (CTD) along the sounding line. A sonic anemometer, mounted on the icebreaker, measured the turbulent heat flux between the air and sea ice surface.

### Reanalysis product dataset and the detection of the Siberian atmospheric river

A gridded atmospheric reanalysis dataset, ERA-Interim, supplied by the European Centre for Medium-Range Weather Forecasts^34^, is used. The reproducibility of reanalysis products over the Arctic without *in situ* shipboard radiosonde observation stations is uncertain, and the values of such datasets differ from those of other products^41^. We verified that the ERA-Interim reproduced the observational results well. ERA-Interim is also used for climatological analyses in the period from 1979 to 2013.

Several methods of detecting atmospheric rivers have been proposed, using both satellite data and reanalysis products^19^. The IVT is also often used as a detector of atmospheric rivers, which are typically detected by identifying contiguous regions ≥ 2000 km in length with IVTs ≥ 250 kg m^−1^ s^−1^ in mid-latitude regions. This study uses the IVT values to identify atmospheric rivers using the same methods as previous studies^34,35^; the horizontal moisture flux multiplied by the specific humidity of the zonal and meridional wind components is integrated from 1000 hPa to 300 hPa. To identify Siberia atmospheric river, we checked the daily mean IVT map during summer season (June, July and August) between 1979 and 2013. This identification shows that the IVT over 200 kg m^−1^ s^−1^ can be used as a threshold for capturing the river-like shape of IVT over the Siberian region, but it is not always promise the atmospheric river. The development of more quantitatively detection is beyond the scope of this paper.

### Numerical simulations

The numerical experiments are based on the polar-optimized version of the Weather Research and Forecasting model^43,44^ version 3.5.1, called the Polar WRF. The simulation area, covering the entire Arctic Ocean and expanding into central Siberia, is set on a 561 × 406 polar stereographic grid with a horizontal grid spacing of 10 km and 69 vertical levels, reaching 50 hPa. The simulation is integrated from 12 UTC on 25 August with 40 second time steps. The initial and lateral boundary conditions are supplied by ERA-Interim, which operated every 6 hour on 37 pressure levels, gridded at 0.75°. Bootstrap sea ice concentration from Nimbus-7 SMMR and DMSP SSM/I-SSMIS^45^ are adopted as the sea ice concentration and boundary condition. The sea ice thickness is fixed at 2 m. The sea surface temperature (SST) is obtained from the National Oceanic and Atmospheric Administration (NOAA) daily Optimum Interpolation SST version 2^46^, which is gridded at 0.25°. The choice of physical parameterization is based on previous literature^47^, but the cumulus parameterization is not used in the simulation.

The experiments were categorized as a control run (CTL), a switching off a latent heat run (DRY), an ice-free run (NoICE), and a reduced Siberian humidity run (RHcut). The CTL was executed under realistic boundary conditions, as shown in the previous paragraph. The DRY turned off an exchange of latent heat through a microphysics after 08 UTC on 29 August at the model time. In this run, the phase change such as a cloud forming occurs by the same manner as in CTL but the latent heat does not released to the atmosphere. The NoICE was executed without the Arctic sea ice in order to observe the influence of the sea ice. The surface boundary condition of the NoICE is that the sea ice for the whole Arctic Ocean was removed, and SST was fixed to the constant value of 4 °C for the area poleward of 65° N. In addition, the air temperature in the initial conditions of the lower troposphere is modified using a simple linear function to

1 $${{\boldsymbol{T}}}_{{\boldsymbol{P}}}=\frac{{{\boldsymbol{T}}}_{{\bf{500}}}-{{\boldsymbol{T}}}_{{\bf{850}}}}{{\bf{350}}}{\rm{\Delta }}{\bf{p}}+{{\boldsymbol{T}}}_{{\bf{850}}}\,$$

Here, T_P_ is the air temperature (°C) at a pressure level P (hPa). T_500_ and T_850_ are the temperatures at 500 hPa and 850 hPa, respectively. ∆p is the pressure difference between 850 hPa to the level of P. This modification is applied from the surface to 850 hPa poleward of 70° N in order to remove the unwanted cold domes from the initial atmospheric conditions. The specific humidity in the NoICE was the same as that of the CTL. The other boundary conditions and the other initial conditions are also the same as those of the CTL.

For the RHcut, the initial condition of the water vapour above central Siberia, westward of 125° E and south of 64° N, was reduced to one-third of the CTL. Furthermore, the moisture advection from the lateral boundary in this region was set to zero. The initial conditions of the water vapour in the other areas, such as over the Arctic Ocean were the same as in the CTL. The other boundary conditions and the other initial conditions are also the same as those of the CTL.

### Trajectory analysis

Backward and forward trajectories of the air parcels were calculated by Read/Interpolate/Plot version 4.5 (http://www.mmm.ucar.edu/wrf/users/docs/ripug.htm). The initial time for the calculation of the trajectories was set to 08 UTC on 29 August. This time was 2 hours later from the actual sounding sample time because the model output at 08 UTC on 29 August was closer to the observation at 06 UTC on 29 August than the model output at the same time. The trajectories of the air parcels initiated at 17 levels (0.13, 0.21, 0.29, 0.38 0.47, 0.59, 0.72, 0.86, 1.03, 1.24, 1.47, 1.75, 2.08, 2.47, 2.92, 3.45, and 4.05 km) over the sounding point were calculated for the preceding 72 hours and the following 36 hours at 30 minute intervals. To evaluate the trajectories of the atmospheric flows from Siberia to the Arctic in RHcut and in CTL, we arranged 500 air parcels at 6 levels (0.5, 1, 2, 3, 4, and 5 km) above central Siberia (small square in Fig. 7c). This area is around the origin of the Siberian route in Fig. 3b, and the trajectories of the parcels of the 108 hours preceding 08 UTC on 26 August were calculated.

### Identification of cold air dome

To identify a cold air dome associated with the sea ice, we assumed that the surface of cold air was characterized by the height of an isentropic surface of 278.4 K because the most of the upper air parcels of the Chukchi Sea route arriving at the observation point were lower than this potential temperature with passing over the observation point (Fig. 3a). Figure 4b shows the horizontal map of time-mean height of the isentropic surface at of 278.4 K estimated by the averaged potential temperature during 06 UTC on 26 and 18 UTC on 31 August. The vertical thermal structures averaged by two regions–one is the whole area of cold air identified by isentropic surface, and the other is the Laptev Sea (quarter sector in Fig. 4b)–are shown in Supplementary Fig. 1b. A strongly stratified layer at about 400 m with large change of relative humidity is seen in the two regions, and they match well to the observed mean structure. Relatively small increase of the potential temperature with height is seen from the surface to at the stably stratified layer formed under the level of 0.5 km for the dry adiabatically, but this layer was saturated. Therefore we can consider this layer as a well- mixed layer for moist adiabatically. The isentropic height surface of 278.4 K averaged in the whole cold air region is located at the nearby the top of the temperature inversion layer (about 0.8 km), which is considered as the interface between the upper free atmosphere and lower Arctic air mass. The isentropic height surface of 278.4 K averaged over the Laptev Sea area is located at near the bottom of the temperature inversion layer, which is the boundary of the warm air advection from outside of the Arctic. These signify that the isentropic surface of 278.4 K can be used as the threshold to roughly distinguish between two air masses.

### Composite analysis

We used daily mean ERA-interim fields during the summer season (Jun, July and August) between 1979 and 2013 for statistics. To make the composite maps averaged for similar events as our observational period, we firstly defined the normalized daily index associated with the moisture injection to the Laptev Sea which is averaged the vertically integrated meridional moisture flux passed among 100° E and 140° E at 75° N (see Fig. 8b). Differences during our observational period exceeded two standard deviations in this definition. Based on this index, we chose the day of anomalous moisture injection which had the highest value around the 5th days with exceedance of one standard deviation. This procedure selected 147 cases in 105 month as the composite members. Finally, we averaged the composite members with 2 day mean and additionally made two kinds of the composite map; one is with 2 days lead and second with 2 days lag. These composite maps depict the transition of synoptic atmospheric condition associated with the anomalous moisture injection events. For remove the global warming signal, the composite members are de-trended at each grid point for long term linear trend estimated by least-squares method in advance.

Additionally we evaluate the sensitivity of the Siberian atmospheric rivers in situations when the sea ice decreased. Firstly we estimated the normalized index associated with the inter-annual variability of the summer time mean sea ice concentration over the coastal area in the Laptev Sea by using the three month mean (June, July and August) Bootstrap sea ice concentration from Nimbus-7 SMMR and DMSP SSM/I-SSMIS. The summer time mean sea ice concentration was defined by the area average squared by 75° N ~ 80° N and 100° E ~ 140° E. Based on this index we divided 147 cases to two groups; positive ice year and negative ice year. The positive ice year contained 73 cases in 19 years (79, 80, 81, 82, 83, 84, 85, 86, 87, 89, 92, 93, 94, 96, 97, 98, 01, 04, 08), and the negative ice year contained 74 cases in 16 years (88, 90, 91, 95, 99, 00, 02, 03, 05, 06, 07, 09, 10, 11, 12, 13). The positive and negative ice year almost corresponded with the past and recent years, respectively. Finally we averaged all the cases for both groups to make composite maps and calculated the anomaly subtracted positive year from negative year. The temperature tendency is also evaluated the difference of the temperature from 2 days before for all cases. The statistical significance is estimated using Student’s *t*-test.

## Electronic supplementary material

Supplementary Information
