# Supplementary material for: Poleward upgliding Siberian atmospheric rivers over sea ice heat up Arctic upper air
Source: Sci Rep. 2018 Feb 13;8:2872. doi: 10.1038/s41598-018-21159-6 (PMC5811560; doi:10.1038/s41598-018-21159-6)
Supplement: Supplementary file 1 — Supplementary Information [file 41598_2018_21159_MOESM1_ESM.docx]

SUPPLEMENTARY INFORMATION

**Poleward upgliding Siberian atmospheric rivers over sea ice heat up Arctic upper air**

Kensuke K. Komatsu^1^, Vladimir A. Alexeev^2^, Irina A. Repina^3^ and Yoshihiro Tachibana^1*^

^1^Faculty of Bioresources, Mie University, Tsu, Japan

^2^International Arctic Research Center, University of Alaska Fairbanks, Fairbanks, USA

^3^Obukhov Institute of Atmospheric Physics, Russian Academy of Sciences, Moscow, Russia

***Corresponding author:** Yoshihiro Tachibana

E-mail: tachi@bio.mie-u.ac.jp

**3^rd^ version**

**Submitted date: 11 Jan. 2018**

**#SREP-17-36772B**


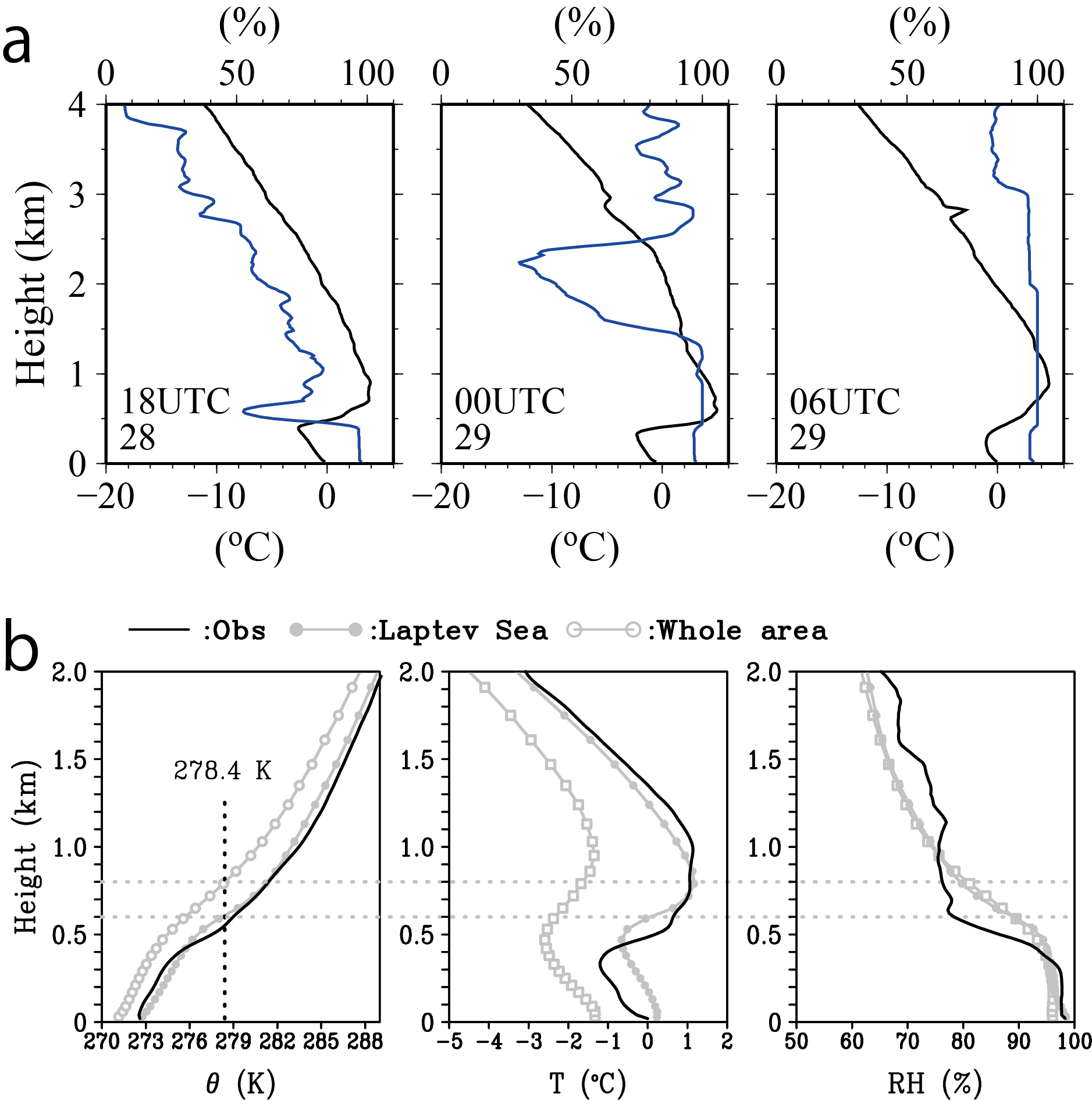


**Supplementary Figure 1.** **Vertical profiles of observed temperatures and relative humidity, and vertical thermal structure averaged over simulated cold air dome.** a) Temperature and relative humidity are shown as black and blue lines, respectively. The left, middle and right panels represent the time evolution of non-trapped cloud tops using strong temperature inversions.

b) The observed and simulated vertical profiles of potential temperature (K), temperature (^o^C), and relative humidity (%) averaged from 06 UTC on 26 to 18 UTC on 31 August. The observation is indicated by black line. The averaged over the simulated cold air dome and the Laptev Sea depicted in Figure 4c are indicated by open and gray circle respectively. Crossed dots show the height about the potential temperature at 278.4 K for simulated value.

**1. Reproducibility of reanalysis product and numerical model**

Supplementary Figure 2 shows the vertical time section of the temperature and specific humidity of ERA-Interim at the time and grid point nearest the radiosonde measurements. Note that the vertical time section in Supplementary Figure 2 is not a ‘snapshot’, but the adopted values seen in this figure change with time to fit the time of the observation. Additionally, the data measured by our radiosonde observations did not correspond to The Global Telecommunication System, indicating that our observational data are not included in ERA-Interim assimilation but are independent of ERA-Interim. Compared with Supplementary Figure 1, the ERA-Interim well reproduced the observational results, including the cold dome on the ice and the humid and warm air mass at 06 UTC on 29 August. The estimation of the integrated water vapour transport (IVT) at 06 UTC on 29 August reached 330 kg m^-1^ s^-1^, and the IVTs at other times agreed well with those observed. We therefore consider ERA-Interim to be a reliable dataset. We compared the observations with other reanalysis products, such as JRA-55, but the reproducibility of the others is not as robust as that of ERA-Interim.

Next, we show the reproducibility of the numerical model. The control (CTL) run well simulated the cyclonic development and its approach towards the sounding points because the surface pressure trends agreed with those observed (Supplementary Figure 3). The cold dome over the sea ice and the warm and humid air masses at altitudes of approximately 1 km on 06 UTC on 29 August were simulated as well. Because the simulated vertical profiles of the air temperature and relative humidity are almost identical to those of the numerical model of the CTL run (see upper central panel of Figure 3a), the numerical model simulation is highly reliable. The IVT reached its maximum value (330 kg m^-1^ s^-1^) at 06 UTC on 29 August, but decreased slightly earlier than the observations showed: the intense moist intrusion in the model passed slightly faster than the actual intrusion, but the model still reproduces the actual humidity conditions, particularly during the former period, which is the focus of this study.


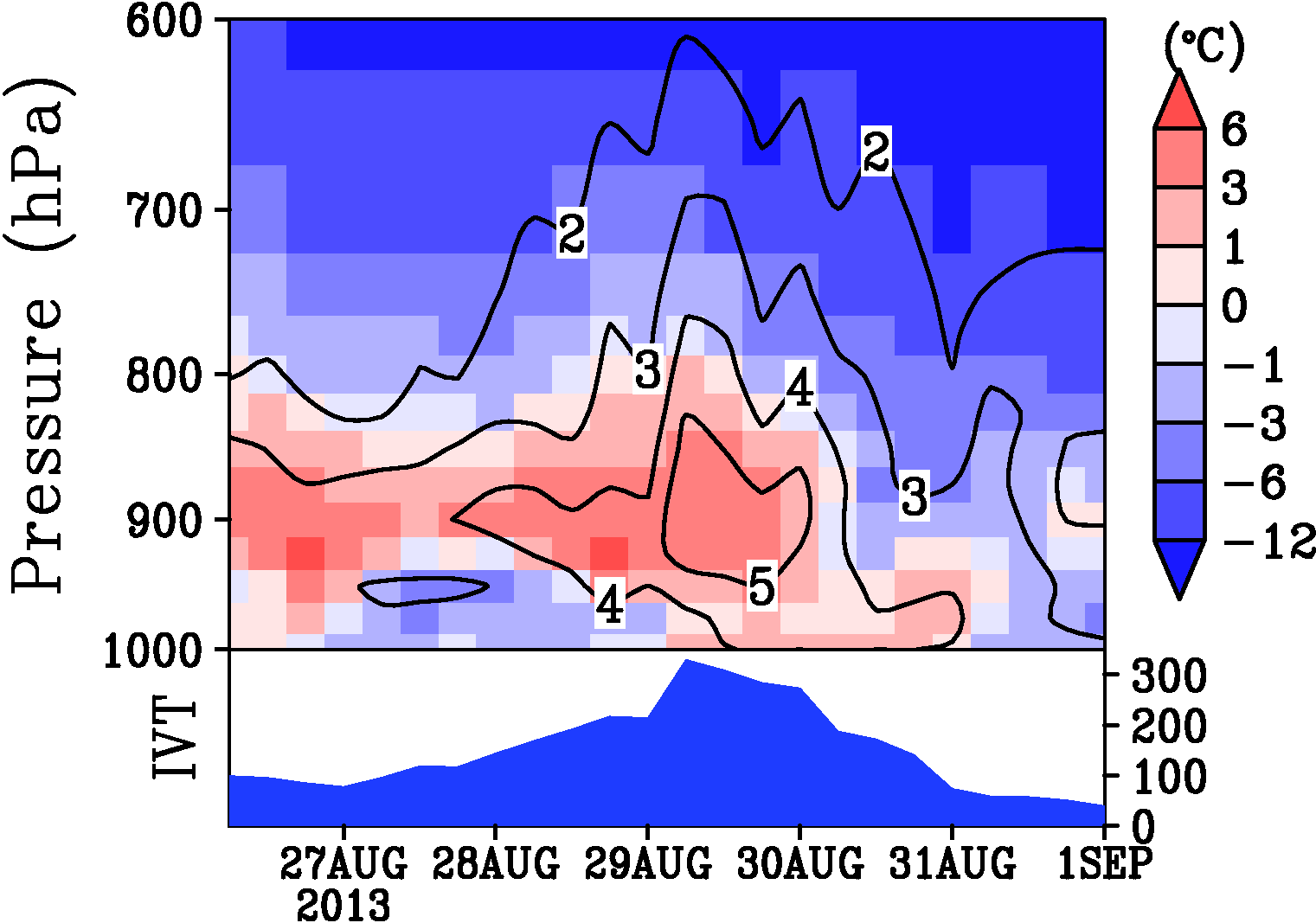


**Supplementary Figure 2.** **Atmospheric vertical time section and IVT values constructed by the reanalysis product.** The atmospheric height-time section indicates temperature (colour, °C) and specific humidity (contours, g kg^-1^). The integrated water vapour transport (IVT) in the atmosphere was estimated to be between 1000 hPa and 300 hPa (kg m^-1^ s^-1^) and is presented in the bottom panel of the atmospheric plot. These are the same as the top and bottom panels in Figure 2b and 2c but for ERA-Interim. The 6 hour data were chosen from the same location and same time as those observed.


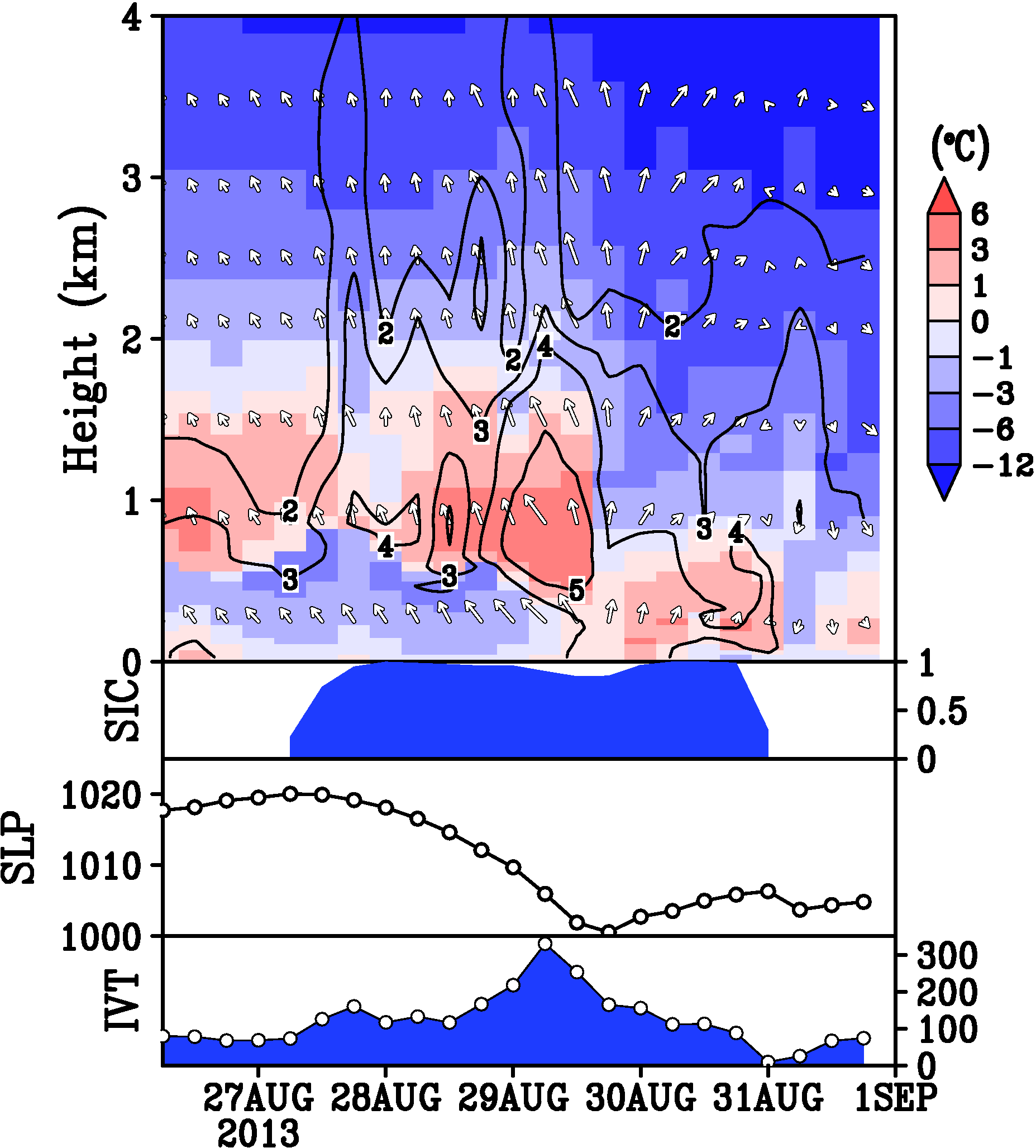


**Supplementary Figure 3. Model-simulated atmospheric vertical time section and sea level pressure.** The atmospheric height-time section indicates temperature (colour, °C), specific humidity (contours, g kg^-1^), and horizontal wind vectors ( m s^-1^). The sea ice concentration (10^2^ %), sea level pressure (hPa), and IVT estimated to be between 1000 hPa and 300 hPa (kg m^-1^ s^-1^) are presented in the bottom panel of the atmospheric plot. These are the same as the top and bottom panels in Figure 2b and 2c but for the CTL run. The 6-hourly data were chosen from the same locations and same times as those observed.
